# Tackling the crystallographic structure determination of the COP9 signalosome

**DOI:** 10.1107/S2059798316001169

**Published:** 2016-03-01

**Authors:** Richard D. Bunker

**Affiliations:** a Friedrich Miescher Institute for Biomedical Research, Maulbeerstasse 66, 4058 Basel, Switzerland; b University of Basel, Petersplatz 10, 4003 Basel, Switzerland

**Keywords:** COP9 signalosome, PCI complexes, low-resolution structure determination, cluster-compound phasing

## Abstract

The COP9 signalosome (CSN) is an essential multi-protein complex in eukaryotes. CSN is a master regulator of intracellular protein degradation, controlling the vast family of cullin–RING ubiquitin (E3) ligases (CRLs). Important in many cellular processes, CSN has prominent roles in DNA repair, cell-cycle control and differentiation. The recent crystal structure of human CSN provides insight into its exquisite regulation and functionality [Lingaraju *et al.* (2014 ▸), *Nature (London)*, **512**, 161–165]. Structure determination was complicated by low-resolution diffraction from crystals affected by twinning and rotational pseudo-symmetry. Crystal instability and non-isomorphism strongly influenced by flash-cooling, radiation damage and difficulty in obtaining heavy-atom derivatives, were overcome. Many different subunits of the same fold class were distinguished at low resolution aided by combinatorial selenomethionine labelling. As an example of how challenging projects can be approached, the structure determination of CSN is described as it unfolded using cluster-compound MIRAS phasing, MR-SAD with electron-density models and cross-crystal averaging exploiting non-isomorphism among unit-cell variants of the same crystal form.

## Abstract 2

The low-resolution structure determination of the COP9 signalosome, a large multi-protein complex, from crystals affected by rotational pseudo-symmetry and twinning is detailed.

## 1. Introduction   

**Figure 1:**
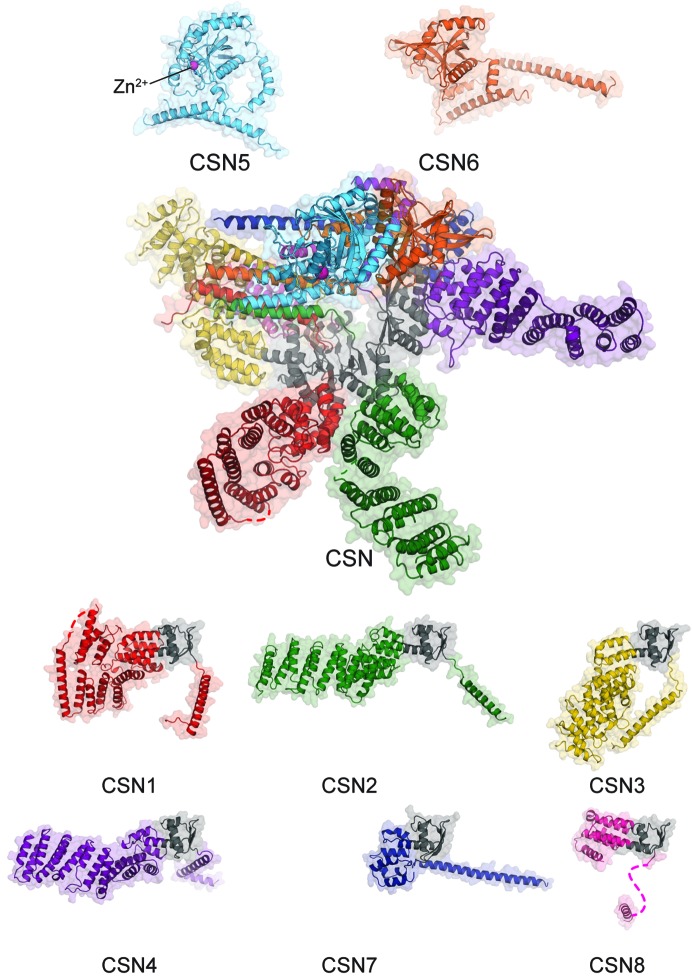
Cartoon representations of the COP9 signalosome complex (centre) and its constituent proteins: the two MPN domain-containing subunits (CSN5 and CSN6; above) and six PCI domain-containing proteins (CSN1–CSN4, CSN7 and CSN8; below). The winged-helix subdomain of each of PCI protein is coloured grey and the Zn^2+^ ion in the active site of CSN5 is labelled. Major loops that were unmodelled because of disorder are shown as dashed lines.

One of a triumvirate of PCI (proteasome lid–COP9 signalosome–initiation factor 3) regulatory complexes, the COP9 signalosome (CSN) is evolutionarily related to the 19S proteosome lid and eukaryotic initiation factor 3. CSN functions enzymatically as a highly regulated isopeptidase that acts exclusively on catalytically activated cullin–RING ubiquitin ligases (CRLs). Human CSN is an assembly of eight proteins, denoted CSN1–CSN8 by decreasing molecular mass, with a total molecular mass of ∼350 kDa (Fig. 1 ▸).

Little structural information for CSN, its constituents or other PCI complexes was available at the start of our work on the human complex. The subunit composition and fold classes were known: CSN1–CSN4, CSN7 and CSN8 contain PCI domains and CSN5 and CSN6 contain MPN (MPR1/PAD1 amino-terminal) domains. PCI domains have an α-helical fold characterized by an array of irregular helical repeats followed by a winged-helix subdomain. MPN domains have a mixed α/β metalloprotease fold. CSN5 is known to be catalytic and to bind a single Zn^2+^ ion.

Crystal structures of parts of four CSN proteins became available in the course of the project: *Arabidopsis thaliana* CSN1 (residues 32–349 of 441; PDB entry 4lct; Lee *et al.*, 2013 ▸), the *Homo sapiens* CSN5 MPN domain (residues 2–257 of 334; PDB entry 4f7o; Echalier *et al.*, 2013 ▸), the MPN domain core of *Drosophila melanogaster* CSN6 (residues 51–184 of 341; PDB entry 4e0q; Zhang *et al.*, 2012 ▸) and *A. thaliana* CSN7 (residues 4–164 of 260; PDB entry 3chm; Dessau *et al.*, 2008 ▸). Crystal structures have been described for paralogues from the 19S proteasome lid (RPN) and initiation factor 3 (elF3) proteins: CSN2 (*D. melanogaster* RPN6, PDB entry 3txn; Pathare *et al.*, 2012 ▸) and CSN8 (human eIF3k, PDB entry 1rz4; *Schizosaccharomyces pombe* RPN12, PDB entry 4b02; Wei *et al.*, 2004 ▸; Sarin *et al.*, 2012 ▸). There were no crystal structures of CSN3 or CSN4. An existing low-resolution electron-microscopy (EM) structure of CSN in negative stain (EMDB entry 1700; Enchev *et al.*, 2010 ▸) proved unhelpful because of substantial differences from the crystal structure. A cryo-EM structure of the CSN–CRL complex in negative stain (EMDB entry 2176; Enchev *et al.*, 2012 ▸), reported after much of the crystallography had been completed, enabled analysis of the general interactions of CSN and a substrate.

## 2. Crystallization, data collection and processing   

Initial microcrystals of full-length human CSN were obtained by hanging-drop vapour diffusion. These were improved to diffraction quality by screening many alternative protein constructs, some of which were identified by mass spectrometry after limited proteolysis. Crystals of one CSN variant containing the canonical isoforms of CSN2–CSN8 and CSN1 isoform 2 (as defined by UniProt) with truncations to the N-termini of CSN1 (51 residues) and CSN5 (11 residues) and the C-terminus of CSN7 (59 residues) formed rhombohedral crystals of 0.1–0.2 µm in diameter that were suitable for X-ray diffraction studies. All crystals analysed were of this construct.

Despite extensive screening, stabilizing conditions for crystals soaking and cryoprotection were not found. Adequate cryoprotection was achieved by transferring each harvested crystal in two steps from 15 to 30%(*v*/*v*) ethylene glycol in a solution formulated to match the crystal-growth solution as closely as possible (artificial mother liquor) before flash-cooling in liquid nitrogen. Soaking experiments with compounds other than tantalum bromide were carried out in artificial mother liquor. Heavy-atom-treated crystals were backsoaked in artificial mother liquor with 15%(*v*/*v*) ethylene glycol for 1–5 min before continuing the cryoprotection protocol as for other crystals.

X-ray diffraction data were collected on beamline X06DA or X10SA at the Swiss Light Source with a Pilatus 2M or 6M detector (Dectris). Diffraction was typically measured with an intense X-ray beam using single-axis rotations (200–360°) with fine slicing (Δφ = 0.05–0.25°) from multiple positions on the crystal. Because the diffraction properties varied substantially from different volumes of the same crystal as well as across crystals, more elaborate data-collection strategies combining multiple data sets were rarely useful. Promising exploration of low X-ray dose high-multiplicity data collection was limited by crystal quality and was not used for analysis. It was only possible to improve the quality of the final data by merging multiple sweeps for the best selenomethionine (SeMet) derivative and a 4 Å resolution refinement data set (crystal c337 mentioned below). Both of these data sets had ∼25-fold average overall multiplicity. Other useful data sets were measured across a single 200–360° rotation.

Diffraction images were processed with *XDS* (Kabsch, 2010 ▸) in multiple passes with geometry parameter recycling. Shadowed regions of the detector were ignored and only the geometrical parameters that refined smoothly to reasonable values across the data set were optimized. Without careful data reduction, small anomalous differences were lost and data-quality issues were exacerbated. Two primary indicators from *XDS* were used to estimate anomalous signal: a mean correlation (Pearson) between two random subsets of anomalous differences (CC_anom_) of ≳0.3 and the mean anomalous difference in units of estimated standard deviation (SigAno) of ≳1.2 for much of the low-resolution range. Crystals containing heavy atoms were sensitive to radiation damage, as indicated by processing statistics suggesting substantial anomalous signal but for which substructure searches failed. Anomalous Patterson maps for damaged crystals were unexpectedly flat.

Several scaling protocols using *XDS*/*XSCALE* and *AIMLESS* (Evans & Murshudov, 2013 ▸) from the *CCP*4 suite (Winn *et al.*, 2011 ▸) were tested with each data set or combination of data sets. After initial phasing, the best as judged by the height and number of log-likelihood gradient (LLG) map peaks found by MR-SAD with *Phaser* (McCoy *et al.*, 2007 ▸) using a common starting model were further analysed. The most successful scaling protocol for the weak anomalous data obtained from SeMet-substituted crystals was to supplement scaling with the *CORRECT* step of *XDS* with scaling with *AIMLESS* using its secondary-beam model without *B*-factor correction. Averaged intensities were converted to structure-factor amplitudes by *TRUNCATE* (French & Wilson, 1978 ▸) for derivative data sets and *XDSCONV* for the refinement data sets, applying a flat prior distribution to twinned data. *R*_free_ sets of reflections were created with twinning and pseudo-symmetry considered using the *PHENIX* suite (Adams *et al.*, 2010 ▸) (also the default in *CCP*4 v.6.4 or newer). Processed diffraction data were analysed with *POINTLESS* (Evans & Murshudov, 2013 ▸) and *phenix.xtriage* from the *PHENIX* suite.

## 3. Crystal characterization   

**Figure 2:**
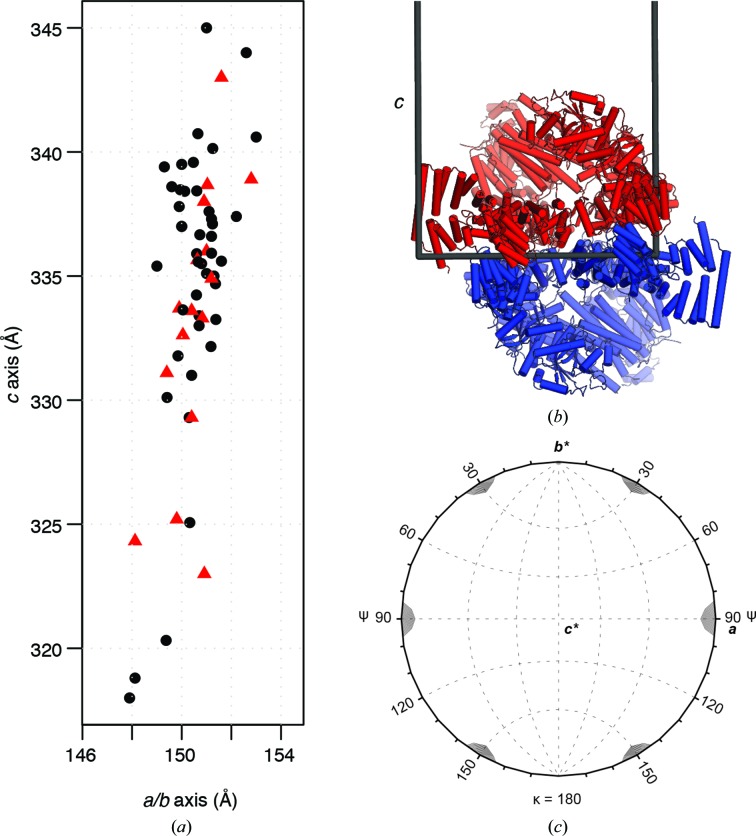
Crystal characterization. (*a*) A scatter plot showing the variation of unit-cell dimensions across 60 CSN diffraction data sets. Crystals with apparent *P*3_1_ or *P*3_2_ space-group symmetry are shown as black circles and crystals with apparent *P*3_1_21 or *P*3_2_21 space-group symmetry are plotted with red triangles. (*b*) The asymmetric unit of a crystal viewed across the *ab* plane showing its two CSN complexes in cartoon mode related by twofold rotational pseudo-symmetry (RPS). The unit-cell axes are shown as solid grey lines. (*c*) Self-rotation function analysis. The κ = 180° section of the native Patterson self-rotation function calculated with data between 6.6 and 20 Å resolution for the untwinned native data set used in MIRAS phasing (space group *P*3_1_), indicating 32 pseudo-symmetry. The peaks are 77% of the height of the origin peak.

In early X-ray testing few crystals diffracted and data to only ∼7 Å resolution were obtained. The unit-cell parameters differed considerably; the length of the *c* axis by 27 Å and that of the *a* and *b* axes by 5 Å (Fig. 2 ▸ *a*). The crystals were clearly trigonal and the pattern of systematic absences indicated a threefold screw axis. Data from most crystals merged best in the enantiomeric space groups *P*3_1_ or *P*3_2_ and had signs of twinning in the *L*-test (Padilla & Yeates, 2003 ▸). Some, however, merged in the higher symmetry space groups *P*3_1_21 or *P*3_2_21, indicated by an *R*_meas_ of 0.06 or lower below ∼10 Å resolution. Cell-content analysis suggested that two or three complexes in the asymmetric unit of the crystal were likely in space group *P*3_1_ or *P*3_2_ (solvent content of ∼66% or ∼50%, respectively) and one complex in the asymmetric unit in space group *P*3_1_21 or *P*3_2_21 (∼66% solvent content).

Self-rotation function analysis showed a twofold rotational axis perpendicular to the crystallographic threefold screw axis for data in space groups *P*3_1_ or *P*3_2_ (Fig. 2 ▸ *c*). Before untwinned data were obtained, we were unable to distinguish whether this twofold rotation was (i) a crystallo­graphic symmetry element generating crystals belonging to space group *P*3_1_21 or *P*3_2_21, (ii) a pseudo-crystallographic noncrystallographic symmetry (NCS) rotation (rotational pseudo-symmetry; RPS), (iii) hemihedral twinning (with twin law *k*, *h*, −*l*) or (iv) a combination of these. All crystals were later established to belong to space group *P*3_1_ with two CSNs in the asymmetric unit (∼66% solvent) and to be affected variably by RPS and twinning. The effects of RPS with twinning compounded by the limited data resolution contributed to the initial uncertainty in the correct space-group assignment.

RPS and twinning introduced different challenges to structure determination. RPS reduced the power of NCS averaging for phase improvement. Reflections related by the twofold NCS axis perpendicular to the threefold crystallo­graphic axis were correlated by RPS such that the magnitude of a given *h*, *k*, *l* is approximately equal to *k*, *h*, −*l*, producing an *R*_merge_ of at most ∼0.43 (crystal c318 mentioned below and deposited as PDB entry 4d18) between these reflections. Because the NCS mates are not completely independent, there is limited alternative sampling of the molecular transform that can be exploited for phase improvement (Kleywegt & Read, 1997 ▸). This was especially notable at low resolution, where the NCS appeared to be crystallographic (Fig. 2 ▸ *c*). Retrospective evaluation with ∼4 Å resolution untwinned data with negligible RPS (crystal c318; PDB entry 4d18), the best quality data for CSN, showed that NCS averaging was beneficial: prime-and-switch density modification of the electron density from refinement of an early model (CC to final map of 0.66) with *RESOLVE* (Terwilliger, 2000 ▸) resulted in a CC of 0.81 with NCS averaging with optimized groups compared with a CC of 0.71 without NCS averaging to the final map.

Twinning introduced more serious problems than RPS into structure determination and most notably encountered when we had difficultly obtaining anomalous data. Unlike twinning in CSN crystals, their RPS does not affect anomalous differences because it does not correlate Bijvoet pairs. Tests with 3.8 Å resolution error-free synthetic data calculated for crystal c343 mentioned below and deposited as PDB entry 4d10 show a twin fraction of 0.36 (twin law *k*, *h*, −*l*), as in the real data, and reduced the mean anomalous difference Fourier peak height for the active-site Zn^2+^ ions by 17%. Despite this, the CSN5 active-site Zn^2+^ ions are found for crystal c343 at 9.5 and 8.5 r.m.s in an anomalous LLG map calculated by *Phaser* (theoretical *f*″ = 2.54 e^−^ at 1.0 Å), demonstrating that anomalous data from twinned CSN crystals could be useful for substructure identification. Twinning also complicated the crystallographic analysis by obscuring the electron density for a conformationally variable subunit (CSN4) and adding a source of non-isomorphism by convoluting the intensity of reflection with a twin that varied across data sets [typically with a twin fraction (α) of between 0.35 and 0.45]. Applying a consistent indexing scheme to highly twinned data was also problematic. Crystallization in a trigonal space group with RPS explains why the crystals formed hemihedral twins with the twin and NCS axes nearly coincident (Lebedev *et al.*, 2006 ▸; Fig. 2 ▸ *b*). Twinning was only modelled in the refinement of c343 by *REFMAC* and was ignored for other procedures and for other data sets.

The finding that twinning was suppressed in crystals grown from seeds enabled untwinned data to be collected. All untwinned data were obtained from seeded crystals. Repeated generations of streak-seeding with a cat whisker were carried out as described previously (Bunker *et al.*, 2012 ▸) adapted to CSN conditions. Beyond its importance for overcoming twinning, seeding was the only technique that consistently improved the diffraction quality of the crystals, eventually allowing data to be collected to ∼4 Å resolution. Cross-seeding from native crystals was needed to grow SeMet-substituted crystals.

Because the phase improvement by NCS averaging was small, we exploited crystal non-isomorphism for phase improvement by cross-crystal averaging, which proved to be crucial for structure solution. We suspected that crystal variability was strongly influenced by handling, but had yet to find a method to control non-isomorphism. Seeking to understand the changes that the crystals underwent before cryogenic data collection (100 K) and to deliberately induce non-isomorphism, room-temperature (RT) diffraction and dehydration experiments using the humidity-control device (HC1; Sanchez-Weatherby *et al.*, 2009 ▸) were carried out at the Diamond Light Source, UK. The unit-cell dimensions at RT were *a* = *b* = ∼152, *c* = ∼344 Å, which are the largest measured for CSN. Dehydration reduced the unit-cell volume, replicating the type of variation found for cryocooled crystals. Although weak, the Bragg peaks at RT were much sharper than at 100 K, suggesting that flash-cooling was detrimental to diffraction. Unfortunately, radiation damage at RT was severe and we were unable to flash-cool dehydrated crystals to enable the collection of sufficient data for analysis.

Returning to the laboratory, we successfully dehydrated crystals in the hanging-drop vapour-diffusion conditions by replacing the well solution with 20% PEG 6000 and equilibrating overnight. This yielded one crystal with a *c* axis of 318 Å (crystal c318; PDB entry 4d18) that diffracted to 4.1 Å resolution. Additive screening yielded a crystal with a *c* axis of 343 Å (c343), which grew spontaneously in a condition supplemented with 10 m*M* urea. Crystal c343, which was partially twinned (twin law *k*, *h*, −*l*; α = 0.36) with RPS, diffracted to 3.8 Å resolution, the highest attained for CSN and provided the other finalized structure (PDB entry 4d10). The extreme unit-cell variants, c318 and c344, were complemented by crystal c337 (with a *c* axis of 337 Å), providing high-multiplicity data to 4 Å resolution. These three non-isomorphous data sets (c318, c337 and c343) became the focus of refinement and model building.

Problems of crystal instability, non-isomorphism and twinning were never completely resolved. Circumventing these issues by screening many crystals, some 1400 crystals were analysed, of which five native crystals, four heavy-atom-soaked derivatives and a redundant set of eight SeMet-substituted derivatives contributed to the two finalized models.

## 4. CSN4   

Having only obtained diffraction data for CSN to ∼4 Å resolution, we expected that model building would be challenging. In parallel, structures of individual subunits to aid the interpretation of the complex were also sought. These attempts produced the structure of human CSN4 on its own with its C-terminal helices truncated and the N-terminal *Strep*-tag II affinity tag retained (PDB entry 4d0p). The diffraction data for initial phasing were collected in-house with a rotating-anode X-ray generator. Structure solution by SIRAS with a mercury derivative and refinement at 1.6 Å resolution was straightforward. Obtaining a high-quality model of CSN4 was fortuitous because the electron density for the N-terminal region of CSN4, the most mobile part of CSN, lacked sufficient detail to model without external structural information.

## 5. Derivatives   

Crystals treated with simple heavy-atom salts did not diffract in early screening or were underivatized. Considering that heavy-atom treatment would be likely to impair the diffraction quality of the crystals and that the asymmetric unit was predicted to contain ∼700 kDa of protein, preparing cluster-compound derivatives was prioritized.

**Figure 3:**
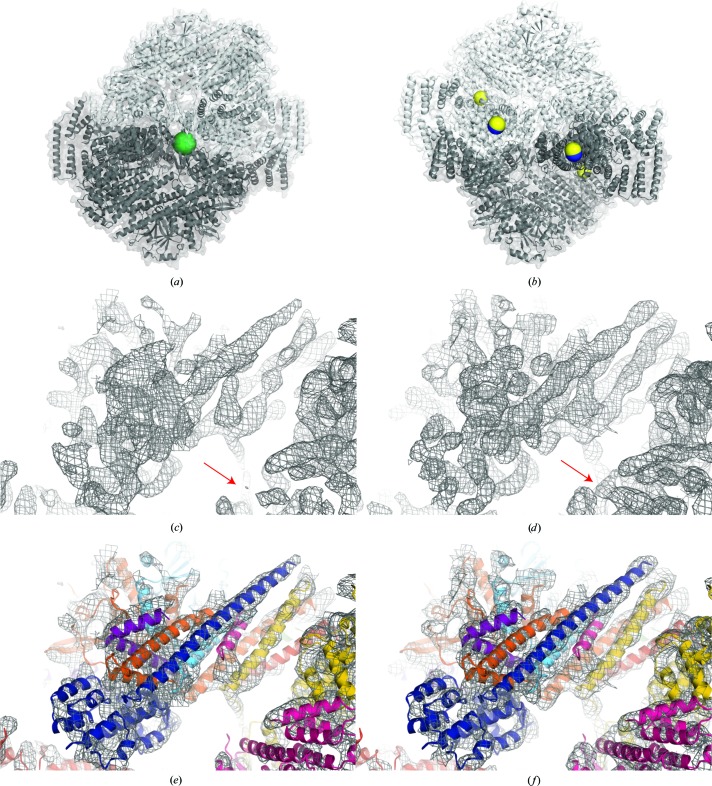
Experimental phasing. (*a*) The asymmetric unit of the single-site tantalum bromide-derivative crystal showing the two NCS copies of CSN in cartoon mode in contrasting shades of grey. The tantalum bromide cluster (green sphere) lies on the NCS axis between the CSNs. (*b*) The same representation as in (*a*) reoriented for clarity showing the deca-ammonium paratungstate cluster (W_12_) positions as spheres in blue for W_12_-I and yellow for W_12_-II, the two correlated derivatives used for MIRAS phasing. (*c*) A segment of the MIRAS-phased experimental electron-density map surrounding CSN7 calculated at 9 Å and contoured at 1 r.m.s.; (*d*) the same segment after solvent flipping with phase extension to 6.6 Å resolution. The red arrows highlight a region improved by density modification. (*e*) The same view as in (*c*) and (*f*) the same view as in (*d*) with the final CSN model embedded in the electron-density map.

After an extensive search, a heavy-atom derivative was produced by incubating a crystal in its original drop for one week with a few grains of tantalum bromide. A single-wavelength anomalous diffraction (SAD) data set was collected for this crystal to 9.5 Å resolution that was not isomorphous to other crystals. Strong anomalous signal was indicated by the processing statistics (overall CC_anom_ of 0.69). Anomalous Patterson analysis revealed a single tantalum bromide position, an unfortunate scenario for SAD phasing (Fig. 3 ▸ *a*). A single-site heavy-atom derivative in a polar space group such as *P*3_1_ or *P*3_2_ is centrosymmetric, which complicates SAD phasing by convoluting the electron density for the crystal with nonrandom ‘noise’ rather than the random ‘noise’ component with a noncentrosymmetric substructure (McCoy & Read, 2010 ▸). Requiring density modification to identify the correct solution and extract electron density for the crystal, this proved fatal and did not advance structure solution.

Continuing the search, a promising derivative was prepared by soaking crystals with deca-ammonium paratungsate (W_12_; Jena Bioscience), a markedly ellipsoidal W_12_ cluster compound. Sites were found with *phenix.hyss* (Adams *et al.*, 2010 ▸) using anomalous data to 9.2 Å resolution after testing a range of low-resolution cutoffs. Finding that the number of W_12_ cluster sites could be manipulated by changes in the crystal-soaking protocol, which is an ineffective approach with tantalum bromide, two derivative crystals suitable for phasing were generated. Two well occupied W_12_ sites were found in a crystal soaked overnight with 0.25 m*M* W_12_ using data collected at the W *L*_III_ absorption peak (W_12_-I; *f*′ = −16, *f*′′ = 24). Four sites, two equivalent to W_12_-I and two unique at ∼20% of the occupancy of the first pair, were found for a crystal soaked overnight with 1 m*M* W_12_ using data collected at the inflection point of the *L*_III_ absorption edge (W_12_-II; *f*′ = −28, *f*′′ = 15). Both W_12_-derivative crystals diffracted to ∼6.6 Å resolution with a mean *I*/σ(*I*) cutoff of 2. W_12_-I had a CC_anom_ of 0.85 and a SigAno of 2.46 and W_12_-II had a CC_anom_ of 0.84 and a SigAno of 2.50 from the low-resolution limit to 9.2 Å, beyond which CC_anom_ dropped precipitously below 0.3.

The two W_12_ positions in each derivative were NCS equivalents wedged between the N-terminal arm of CSN2 and CSN8 in a neighbouring asymmetric unit. The two weaker sites in W_12_-II were NCS equivalents nestled beside CSN3, although neither NCS relationship was recognized until the election density improved after initial phasing (Fig. 3 ▸ *b*).

**Figure 4:**
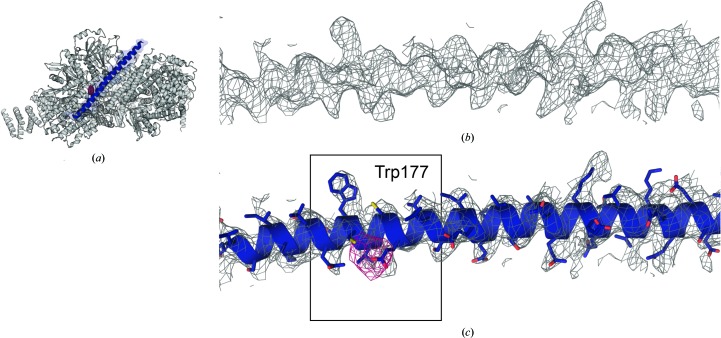
Sequence assignment and validation of the 50-residue (70 Å long) CSN7 C-terminal helix. (*a*) The location of the helix (highlighted in blue) in CSN (white in cartoon mode). The initial sequence assignment was anchored on a prominent side-chain bump for Trp177 in an electron-density map calculated by cross-crystal averaging across the three refinement data sets for crystal c343 with *DMMULTI* at 4.0 Å resolution prior to side-chain modelling.. (*b*) A segment of this electron-density map (grey mesh) contoured at 1 r.m.s. with a radius of 4 Å around the CSN7 C-terminal helix. (*c*) The same view as in (*b*) with the final model embedded (shown in cartoon mode with side chains in blue). The sequence registered was confirmed with a peak found for a modified Cys residue neighbouring Trp177 in an anomalous LLG map (pink mesh; peak height of 6.3 r.m.s.d.) calculated for an Hg-derivative crystal. Additionally, Trp177 has been identified biochemically as crucial for the interaction of CSN7 with CSN6 (Kotiguda *et al.*, 2012 ▸). The side chain of Trp177 of CSN7 is buried in a hydrophobic pocket created by CSN6 in CSN, explaining the biochemical findings.

Substructures were unable to be found for many data sets from heavy-atom-soaked crystals. These were revisited as the structure improved with MR-SAD using *Phaser* and only one, a mercury derivative prepared with *p*-chloromercurybenzoic acid, yielded a heavy-atom substructure. This derivative, which diffracted to 7.5 Å resolution, confirmed the location of 21 cysteine residues in the model (Fig. 4 ▸).

## 6. Initial phasing   

Combinations of native and derivative data sets were evaluated for their potential for phasing using the analysis steps of *autoSHARP* (Vonrhein *et al.*, 2005 ▸) guided by criteria outlined previously (Rudenko *et al.*, 2003 ▸).

The two W_12_-cluster derivatives were combined with an isomorphous, untwinned native data set for MIRAS phasing. After scaling, W_12_-I and W_12_-II had unweighted cross-crystal *R* factors on amplitudes to the native data set of 0.183 and 0.173, respectively, and an *R* factor of 0.135 to each other (in the full resolution range 50–6.6 Å). The cross-crystal weighted *R* factor to the native data set was larger than the *R* factor across the resolution range 50–8.7 Å for both derivatives. Phasing was initiated with *SHARP* (Bricogne *et al.*, 2003 ▸) with one of the two sites found for W_12_-I, using LLG map peaks for its second site to confirm the validity of both. The heavy-atom model was completed for both derivatives by iterative interpretation of the LLG maps first as single atoms and then as spherically averaged descriptions of W_12_. The global non-isomorphism parameters (NISO_BGLO and NANO_BGLO in *SHARP*) were unrefined in cycles with an incomplete heavy-atom model. To avoid correlated non-isomorphism in *SHARP* encountered here with two similar derivative crystals, which can lead to overestimated phase quality, the better of the two derivatives, W_12_-II, was set as the reference for phasing.

The peaks in the LLG maps for the major sites were ellipsoidal, suggesting that the cluster had a preferred orientation for binding. Approaches aimed at extending the phased resolution beyond ∼9 Å by modelling the fine features of the W_12_ sites by placing two or three sites closely together in the LLG map peaks, reducing the radius of the cluster for spherical averaging or fitting the W_12_ framework of the cluster as a rigid body were of little benefit. The best phases to 9 Å resolution were obtained from a single spherically averaged W_12_ at each position with a slightly smaller radius (4.5 Å) than expected from its crystal structure (∼4.75 Å).

After density modification by solvent flipping using *SOLOMON* (Abrahams & Leslie, 1996 ▸) operated through its interface in *SHARP*, the correct hand of the substructure was readily discriminated by the presence of bundles of tubular density, indicative of α-helices, in space group *P*3_1_ and fragmented electron density for space group *P*3_2_ (Fig. 3 ▸).

Although little phase information was derived by NCS averaging, it was still undertaken. The NCS twofold operator was identified in the *SOLOMON* map with *phenix.find_ncs_from_density* (Terwilliger, 2013 ▸) and averaged with *RESOLVE*. Density modification with *SOLOMON* and averaging with *RESOLVE* generated phases to 6.6 Å resolution.

As the primary source of structural information, an effort was made to generate the best possible electron-density map for subsequent structure-determination steps. Throughout initial phasing, different phase sets were assessed by comparing the peak height in anomalous difference Fourier maps calculated for the tantalum bromide derivative. This gave an indication of the phase error independent of the figure of merit (FOM) and other internal estimates phase of quality. Theoretically, FOM is the mean cosine of the phase error, but in practice the FOM calculated from phase probabilities generally underestimates their error and can be unreliable for ranking phase quality (Pannu *et al.*, 2003 ▸).

## 7. Subunit identification and selenomethionine phasing   

Owing to the similarity within the two subunit families (the six PCI subunits and two MPN subunits), the splayed structure of CSN and its crystal-packing interactions, it was not possible to define the two biologically relevant complexes in the asymmetric unit from the initial low-resolution electron density.

We produced CSN recombinantly in insect cells using separate viruses for each subunit. To identify the individual subunits we exploited the remarkable permissibility of CSN assembly, which allowed purification of the complete complex from complementary subassemblies combined at lysis. Several selectively substituted SeMet derivatives were prepared and the Se sites found for these crystals were used to define its subunit arrangement. This was coupled with attempts to generate phase information to improve the electron density; hence, we also performed combinatorial labelling of subunits in an effort to provide adequate signal for phasing. Crystals of four derivatives with single SeMet protein substitutions (CSN1, CSN2, CSN3 and CSN8) and four combinatorial derivatives (CSN1 and CSN4, CSN2 and CSN4, CSN2, CSN3, CSN5 and CSN8, and CSN2, CSN3, CSN5, CSN6, CSN7 and CSN8) were obtained.

A sphere of electron density containing the approximate volume of an asymmetric unit was extracted from the map from initial phasing and prepared as a search model with *phenix.cutout_density*. The electron density was distributed to the SeMet-derivative crystals by MR or rigid-body optimization with *Phaser*. Protocols detailing the use of density maps as search models in MR with *Phaser* are given in Jackson *et al.* (2015 ▸). The positioned electron density was used to initiate an anomalous substructure search using the MR-SAD phasing procedure in *Phaser*. The electron density was improved by cross-crystal averaging with *phenix.multi_crystal_average* combining the current best Se-SAD phases and MIRAS phases with two non-isomorphous native data sets. The process was then repeated starting with the improved electron-density map.

LLG map peaks higher than 6 r.m.s.d. (root-mean-square deviations above the mean) were assigned as a heavy atoms automatically by *Phaser*. It was also beneficial to inspect the LLG maps manually and designate peaks above ∼4 r.m.s.d. as heavy atoms if they were found for multiple crystals. Two Zn^2+^ ions, one for each CSN5, were located in the derivatives and confirmed by analysing native data. After three iterations this process converged, having found 82 of the 176 Se sites expected from the number of methionine residues in the crystallized construct.

Most selenium positions were found with derivatives of all subunits except CSN1 and CSN4 labelled with Se, mostly from one exceptional crystal that diffracted to 4.8 Å resolution (providing phases to ∼8.0 Å resolution based on a 0.3 FOM cutoff). The other derivatives were substantially poorer quality but were crucial for identifying the subunits. From these sites each subunit was identified and the biologically relevant asymmetric unit was defined.

## 8. Initial model building   

An initial polyalanine model was built interactively with *Coot* (Emsley *et al.*, 2010 ▸) into the best electron-density map after subunit identification and selenomethionine phasing (6 Å resolution). Portions of the models of human CSN4 and CSN5, structures of the CSN1, CSN6 and CSN7 orthologues, and homology models of CSN2 and CSN8 were docked into the density manually guided by the ensemble of Se sites and secondary-structure predictions from *PSIPRED* (Buchan *et al.*, 2013 ▸). Homology models were prepared with *Phyre*2 (Kelley *et al.*, 2015 ▸) and *I-TASSER* (Zhang, 2007 ▸). Ideal α-helices were placed in tubular electron density not described by model fragments.

At this stage, many refinement and density-modification approaches were trialled in an attempt to improve the interpretability of the electron density. The strategy that emerged as being successful was to repeatedly extend and rebuild the unrefined model into electron density from refinement with *autoBUSTER* (Bricogne *et al.*, 2011 ▸) against the 4 Å resolution untwinned data from crystal c337 and subsequent *RESOLVE* prime-and-switch density modification with NCS averaging. Initially, the major fragments were optimized as rigid bodies. Missing-atom modelling was enabled using the cross-crystal averaged phases from subunit identification in refinement and to define the distribution of unmodelled atoms. In later cycles the experimental phases were abandoned and it became advantageous to include positional refinement with local structure-similarity restraints (LSSR) (Smart *et al.*, 2012 ▸) between NCS mates and to the high-resolution models of human CSN4 and CSN5 (PDB entry 4f7o).

Model building was continued in *Coot*, with tight restraints on geometry, Ramachandran plot and appropriate secondary structure. This procedure plateaued with a fragmented model that was approximately 50% complete and was unable to provide sufficient phase information to enable its completion.

## 9. Model completion   

A polyserine version of the model from initial building was distributed to two non-isomorphous native crystals (c318 and c343) by rigid-body refinement with one group per subunit using *autoBUSTER*. Because large-scale domain movements within CSN accompanying the unit-cell changes placed the models beyond the radius of convergence of conventional refinement, the electron density from rigid-body fitting or subsequent refinement attempts was uninformative. Good-quality electron density revealing the conformational differences in c318 and c343 was, however, generated by following rigid-body fitting with deformable inelastic network refinement (DEN) with *CNS* (Schröder *et al.*, 2010 ▸). DEN was performed with restraints to the input model using tight NCS restraints and parameter optimization as recommended in Schröder *et al.* (2014 ▸). The conformation of CSN4 was shown by DEN to vary considerably across crystals and between NCS mates and enabled a more effective rigid-body decomposition to be defined for electron-density averaging and refinement. Rigid domains were identified manually or with *DynDom*3*D* (Poornam *et al.*, 2009 ▸). However, the DEN models were highly distorted and were therefore replaced with earlier models before proceeding.

The model was then extended and refined iteratively across the three non-isomorphous unit-cell variants (c318, c337 and c343). The untwinned structures, c318 and c337, were refined using *autoBUSTER* with reference-model restraints applied as LSSR. The twinned structure, c343, was refined by *REFMAC* (Murshudov *et al.*, 2011 ▸) with reference-model restraints generated by *PROSMART* (Nicholls *et al.*, 2012 ▸). Co-refinement was carried out using reference-model restraints generated across crystals, typically between crystals c318 and c337 and between crystals c337 and c343, to parts of the model lacking restraints to high-resolution CSN4 and CSN5 models. Reference model restraints were applied loosely (setting ‘-target_weight’ to 0.5 for *autoBUSTER* and ‘external weight scale’ to 10 for *REFMAC*) from the second round of refinement. This straightforward strategy allowed the three structures to be compared readily and facilitated cross-crystal averaging. It also stabilized refinement by improving the observation-to-parameter ratio, as judged by a narrowing of the *R*_free_–*R*_work_ gap, and prevented unwarranted divergence among the models. By allowing comparison with the untwinned forms, co-refinement provided an internal control for model bias in twin refinement of c343.

Maximum-likelihood twin refinement of c343 by *REFMAC* proved to provide the most interpretable electron density for model building. Modelling the twin only subtly improved the quality of the electron-density maps, most obviously suppressing peaks in solvent regions but crucially also increasing the clarity of some side chains.

**Figure 5:**
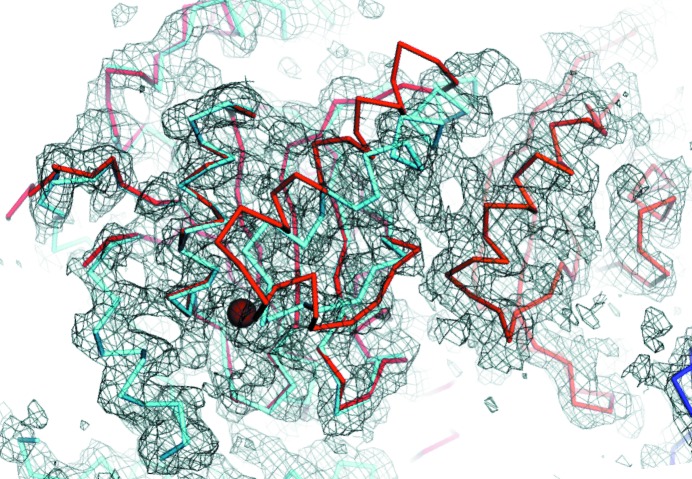
Representative electron density for CSN. A portion of the electron-density map (grey mesh) surrounding the active site of CSN5 (cyan) calculated at 4 Å resolution and contoured at 1 r.m.s. for crystal c337 by cross-crystal averaging with NCS averaging across the three non-isomorphous refinement data sets with *RESOLVE*. The structure-factor amplitudes were sharpened by −134 Å^2^ (isotropic *B* factor 40 Å^2^) before density modification. C^α^ traces are shown for CSN5 (cyan) superposed with the high-resolution structure of CSN5 (red; PDB entry 4f7o) and CSN6 (orange). CSN5 assumes an auto-inhibited conformation in CSN, which was not found for the crystal of the CSN5 fragment on its own. This results in conformational differences at the active site (indicated by a red sphere for the active-site Zn^2+^ ion) of the two CSN5 models.

Applying the rigid bodies identified by DEN earlier, NCS-averaged prime-and-switch density-modified versions of the maps from refinement were generated with *RESOLVE*. These maps were averaged across crystals again with NCS averaging using *phenix.multi_crystal_average* (or *RESOLVE* directly) and *DMMULTI* from the *CCP*4 suite and were used for model building with the electron density from refinement. The final averaging procedure was performed among the three non-isomorphous refinement data sets and the high-resolution CSN4 structure with CSN partitioned into 12 domains (Fig. 5 ▸). *B*-factor sharpening of the structure-factor amplitudes before density modification and map coefficients from refinement was crucial to enhance the electron density for side chains.

Supplemental restraints to preserve the geometry of the CSN5 active-site Zn^2+^ ion and secondary structure, including intermolecular hydrogen bonds across the β-sheet formed by the PCI subunits, were defined manually for *autoBUSTER* and *REFMAC*. TLS refinement was applied with the same rigid bodies as NCS averaging.

Near the completion of c343, *PDB_REDO* (Joosten *et al.*, 2012 ▸) locally modified to include external structural restraints and suitable TLS, jelly-body and twin-refinement parameters was used to derive optimized weights for positional and *B*-factor refinement, solvent-masking parameters and to carry out paired refinement (Karplus & Diederichs, 2012 ▸), establishing a high-resolution limit of 3.8 Å.

For the final cycles of refinement of c318 with *autoBUSTER* an optimal X-ray weight, which was poorly determined with low-resolution data with NCS and had been fixed earlier, *B*-factor refinement scheme and through-bond *B*-factor correlation weight were selected after testing a range of values in refinement and assessing the results by *R* factor, LLG_free_ and geometric validation with *MolProbity* (Chen *et al.*, 2010 ▸). Paired refinement of c318 was carried out by *autoBUSTER* with *R* factors calculated using *REFMAC*, establishing a high-resolution limit of 4.08 Å.

The resolution cutoffs for the refinement structures correspond to a CC_1/2_ of ∼0.3 in a 0.1 Å wide outermost shell for the refinement data sets. This is roughly 0.2 Å beyond the resolution at which *I*/σ(*I*) drops to 2, a suggested starting point for revising the high-resolution limit from former standards (Luo *et al.*, 2014 ▸).

The models were finalized for c318 and c343, the two most extreme unit-cell variants. Refinement and data processing statistics for these structures, CSN4, data-processing statistics for c337 and the MIRAS phasing data sets, and phasing statistics have been published elsewhere (Lingaraju *et al.*, 2014 ▸).

## 10. Conclusions   

The structure determination of CSN was difficult, lacking a major breakthrough to propel it to rapid completion. Progress was incremental, with regular sanity checks required to convince us that we were moving forward. Structure solution began to look feasible after the collection of the three non-isomorphous native data sets of substantially better quality than the others, diffracting to the bounds of interpretable resolution (∼4 Å). Initial phasing and the substructure searches were re-initiated several times using an electron-density map or a coordinate model as the prior distribution. Model building and refinement was conservative, repeatedly returning to unrefined structures to limit model bias until a successful strategy was developed to enable completion. The final structure, however, rewarded the effort by providing substantial insight into the regulation and activity of CSN and the architecture of PCI complexes.

Difficulties in crystallographic analysis can be traced to the dramatic conformational variation of CSN4. CSN4 only forms crystal contacts in the small unit cell of crystal c318. This variation broke the symmetry that would have made the NCS crystallographic. Had the NCS been crystallographic the crystals would not have been able to twin in the same manner. Crystal contacts stabilizing CSN4 would have potentially increased the diffraction resolution, removed unit-cell variability and enabled derivatives to be found more easily. Crucially, however, the conformational variability of CSN4, which is involved in the substrate recognition and catalytic activation of CSN, is functionally important. Avoiding these crystallographic issues by pursuing the structure of CSN with an N-terminally truncated CSN4 would have been less meaningful and our functional insight would potentially have been more limited.

The strengths of the *CCP*4, *CNS*, Global Phasing and *PHENIX* crystallographic software packages were harnessed for structure determination, which would not have been possible without the combination of methods. At several points in structure solution we encountered deficiencies in the current methods for experimental phasing, particularly in the treatment of cluster-compound derivatives. The co-refinement approach used here was inspired by an implementation in *MAIN* (Turk, 2013 ▸). Proposed methods extending this to simultaneous cross-crystal refinement with electron-density averaging (Brunger, 2005 ▸; Nicholls *et al.*, 2012 ▸) would have been beneficial for CSN and likely other challenging structures.
